# Blood taken immediately after fatal resuscitation attempts yields higher quality DNA for genetic studies as compared to autopsy samples

**DOI:** 10.1007/s00414-023-02966-7

**Published:** 2023-02-11

**Authors:** Caroline Stanasiuk, Hendrik Milting, Sören Homm, Jan Persson, Lars Holtz, Axel Wittmer, Henrik Fox, Thorsten Laser, Ralph Knöll, Greta Marie Pohl, Lech Paluszkiewicz, Thomas Jakob, Bernd Bachmann-Mennenga, Dietrich Henzler, Steffen Grautoff, Gunter Veit, Karin Klingel, Erika Hori, Udo Kellner, Bernd Karger, Stefanie Schlepper, Heidi Pfeiffer, Jan Gummert, Anna Gärtner, Jens Tiesmeier

**Affiliations:** 1 Erich and Hanna Klessmann-Institute for Cardiovascular Research and Development, Heart- and Diabetes Center NRW, University Hospital of the Ruhr-University Bochum, Georgstr. 11, D-32545 Bad Oeynhausen, Germany; 2 Institute for Anesthesiology, Intensive Care- and Emergency Medicine, Johannes Wesling Hospital Minden, MKK-Hospital, Campus OWL, Ruhr-University Bochum, Bochum, Germany; 3 Emergency Department, Herford Hospital, Campus OWL, Ruhr-University Bochum, Bochum, Germany; 4 Institute for Pathology, Herford Hospital, Campus OWL, Ruhr-University Bochum, Bochum, Germany; 5 Clinic for Thoracic and Cardiovascular Surgery, Heart- and Diabetes Center NRW, D-32545 Bad Oeynhausen, University Hospital of the Ruhr-University Bochum, Bochum, Germany; 6 Center for Congenital Heart Diseases, Heart and Diabetes Center NRW, 32545 Bad Oeynhausen, University Hospital of the Ruhr-University Bochum, Bochum, Germany; 7 Karolinska Institute, University Hospital, Myocardial Genetic, 14157 Huddinge, Sweden; 8 Intensive Care and Emergency Medicine, Herford Hospital, Campus OWL, Ruhr-University Bochum, University Clinic for Anesthesiology, Bochum, Germany; 9 Institute for Pathology and Neuropathology, University Hospital Tuebingen, D-72076 Tuebingen, Germany; 10 Institute for Pathology, Johannes Wesling Hospital Minden, MKK-Hospital, D-32429 Minden, Campus OWL, Ruhr-University Bochum, Bochum, Germany; 11 Institute for Forensic Medicine, University Hospital, Wilhelms-University Muenster, Muenster, Germany; 12 Institute for Anesthesiology, Intensive Care- and Emergency Medicine, Luebbecke MKK-Hospital, Campus OWL, Ruhr-University Bochum, Bochum, Germany; 13 Present address: Clinic for Anesthesiology, Intensive Care Medicine, Emergency Medicine and Pain Medicine, Bielefeld Hospital, University Hospital Eastern Westphalia–Lippe, Bielefeld University, Bielefeld, Germany

**Keywords:** Molecular autopsy, Resuscitation, Next-generation sequencing, Cardiomyopathy, Channelopathy, Emergency medical service, DNA stability

## Abstract

**Background:**

The out-of-hospital cardiac arrest (OHCA) in the young may be associated with a genetic predisposition which is relevant even for genetic counseling of relatives. The identification of genetic variants depends on the availability of intact genomic DNA. DNA from autopsy may be not available due to low autopsy frequencies or not suitable for high-throughput DNA sequencing (NGS). The emergency medical service (EMS) plays an important role to save biomaterial for subsequent molecular autopsy. It is not known whether the DNA integrity of samples collected by the EMS is better suited for NGS than autopsy specimens.

**Material and methods:**

DNA integrity was analyzed by standardized protocols. Fourteen blood samples collected by the EMS and biomaterials from autopsy were compared. We collected 172 autopsy samples from different tissues and blood with postmortem intervals of 14–168 h. For comparison, DNA integrity derived from blood stored under experimental conditions was checked against autopsy blood after different time intervals.

**Results:**

DNA integrity and extraction yield were higher in EMS blood compared to any autopsy tissue. DNA stability in autopsy specimens was highly variable and had unpredictable quality. In contrast, collecting blood samples by the EMS is feasible and delivered comparably the highest DNA integrity.

**Conclusions:**

Isolation yield and DNA integrity from blood samples collected by the EMS is superior in comparison to autopsy specimens. DNA from blood samples collected by the EMS on scene is stable at room temperature or even for days at 4 °C. We conclude that the EMS personnel should always save a blood sample of young fatal OHCA cases died on scene to enable subsequent genetic analysis.

**Supplementary Information:**

The online version contains supplementary material available at 10.1007/s00414-023-02966-7.

## Background

Every year in the European population, 67–170 out-of-hospital cardiac arrest (OHCA) cases per 100,000 are reported. Of those, 19–97 per 100,000 receive out-of-hospital resuscitation attempts (OHRAs) by the emergency medical services (EMS) [1]. Among these patients of all age groups unrecognized cardiac conditions such as coronary heart disease, cardiomyopathies or different arrhythmogenic diseases may lead to sudden cardiac death (SCD) [2]. However, the incidence of these conditions is not precisely known for several reasons [3].

Especially in the young, SCD is associated with higher proportion of arrhythmogenic or structural heart diseases, which may be related to genetic predispositions [4, 5]. However, the identification of genetic forms of SCD needs next-generation sequencing technologies (NGSs) which can only be applied if non-degraded biological material for extraction of high-quality DNA is available [6, 7]. The combination of autopsy and genetic analysis of fatal OHCA cases allows the identification of genetic forms of SCD in the young [5]. Due to low autopsy frequencies in several countries, a high proportion of these cases remain unexamined (see for an overview [4, 8]). This may have consequences on health of others in affected families [2, 4, 8]. Moreover, biological materials for the isolation of genomic DNA (gDNA) may be not suitable for high-throughput DNA sequencing because of postmortem degradation processes [9, 10] leading to fragmented gDNA at autopsy [11–14].

The recent European Resuscitation Council guidelines from 2021 (ERC-2021) do not consider the collection of blood samples from fatal OHCA cases by the EMS on scene. Instead, it is recommended to collect biomaterial from autopsy [15]. However, the minority of OHCA cases are autopsied, and autopsy biomaterial may be not suitable for NGS. Especially formalin-fixed paraffin-embedded (FFPE) tissue will result in comparably low-quality sequencing results. For these reasons, blood samples from fatal OHCA cases collected by the EMS on scene may play a deciding role to allow at least molecular autopsy by specialized cardiogenetic services if autopsies are not performed [16].

**Fig. 1:**
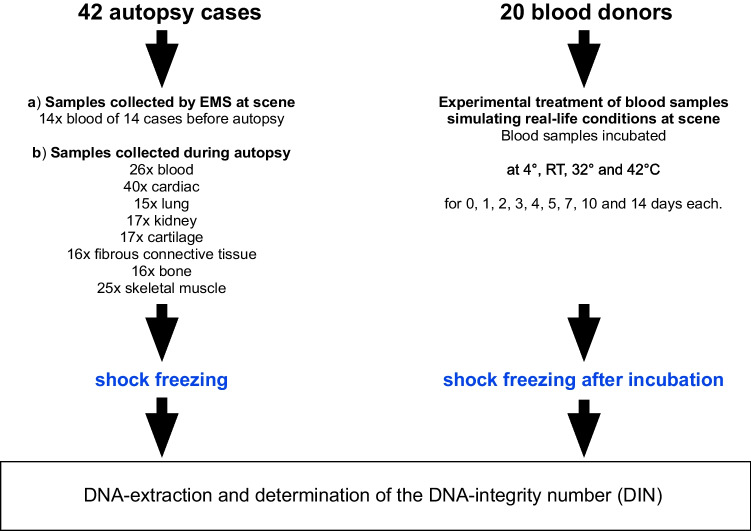
Study overview (compare also Table 1 for details)

In this study, we determined the DNA integrity number (DIN) of the gDNA isolated from autopsy specimens and compared the data to gDNA extracted from blood collected by the EMS (Fig. 1). Moreover, we investigated different storage conditions for the blood samples with later gDNA extraction to simulate real-life conditions for handling blood samples by the EMS.

## Material and methods

### Study cohort

**Fig. 2:**
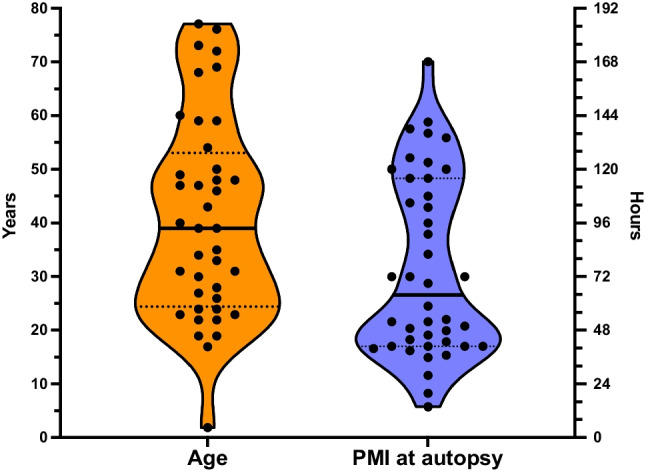
Violin plot of age distribution and postmortem intervals (PMIs) at autopsy. The median age of autopsy cases is 39 years. The median PMI was 64 h. Truncated violin plots showing medians as solid lines, quartiles are given as dotted lines

We included in total 42 hospital and forensic autopsy cases of deceased individuals aged 2–76 years. In total, 172 samples from hospital and forensic autopsies derived from heart (*n* = 40), lung (*n* = 15), kidney (*n* = 17), cartilage (*n* = 17), fibrous connective tissue (*n* = 16), bone (*n* = 16), skeletal muscle (*n* = 25), and blood (*n* = 26) were collected. Samples were taken at postmortem intervals (PMIs) between 14 and 168 h and stored at − 80 °C until gDNA extraction. All but one autopsy case was cooled until examination. For comparison, the EMS collected blood samples from 14 fatal OHCA cases at scene who were later autopsied were included (for baseline data, see Table 1, Figs. 1 and 2). For experiments simulating real-life conditions of blood collection by the EMS, we have also included samples of 20 anonymous blood donors.

**Table 1:** Baseline data of autopsy cases

Case no.	Age [years]	Sex	Cause of death	Cooled until autopsy	PMI [h]	Biomaterials								
						H	L	K	C	FC	B	SKM	BL-A	BL-EMS
#01	47	M	Cardiac decompensation	Yes	14	X						X		X
#02	59	M	Heart failure	Yes	20	X						X	X	
#03	40	M	Heart failure	Yes	28	X						X		X
#04	23	M	Global respiratory decompensation with ARVC	Yes	36		X	X	X	X	X		X	
#05	72	F	N.A.	Yes	37	X								
#06	48	M	Heart failure	Yes	39	X								X
#07	39	F	Respiratory decompensation with multiple organ failure	Yes	40	X	X	X	X	X	X		X	
#08	30	M	N.A.	Yes	41	X						X		X
#09	50	F	Acute recurrent myocardial infarction and acute cerebral mass hemorrhage	Yes	41	X	X	X	X	X	X		X	
#10	43	M	Acute cardiac decompensation in left ventricular myocardial infarction	Yes	41	X	X	X	X	X	X	X	X	
#11	24	F	N.A.	Yes	41	X						X	X	
#12	49	M	N.A.	Yes	43	X					X	X	X	
#13	60	M	Acute global heart failure in pulmonary artery embolism and acute pancreatitis	Yes	44	X	X	X	X	X	X		X	
#14	76	M	Multiple organ failure	Yes	46	X	X	X	X	X	X		X	
#15	46	M	N.A.	Yes	48	X						X		
#16	77	F	Acute heart failure with pericardial tamponade and hemothorax	Yes	49	X	X	X	X	X	X		X	
#17	19	M	N.A.	Yes	50	X						X		X
#18	59	M	N.A.	Yes	52	X						X		X
#19	N.A.	N.A.	N.A.	Yes	52	X	X	X	X	X	X		X	
#20	35	F	N.A.	Yes	53	X			X	X		X	X	
#21	2	M	N.A.	Yes	59	X			X			X	X	
#22	N.A.	F	Acute cardiac decompensation with multiple organ failure	Yes	69	X	X	X	X	X	X		X	
#23	31	F	N.A.	Yes	72	X			X			X	X	
#24	48	M	N.A.	Yes	72	X						X		X
#25	24	M	N.A.	Yes	72	X								X
#26	31	F	Perforated aneurysm of the ascending aorta	Yes	82	X						X	X	X
#27	33	F	N.A.	No	91	X						X		X
#28	39	F	N.A.	Yes	96			X					X	
#29	69	F	Multiple organ failure in fungal sepsis	Yes	103	X	X	X	X	X	X		X	
#30	47	M	Cardiac decompensation	Yes	105	X	X	X	X	X	X		X	
#31	17	F	Not clear	Yes	108	X						X		
#32	54	M	Combination of hemorrhagic shock and right heart failure	Yes	116	X	X	X		X	X		X	
#33	28	M	Acute left heart decompensation with myocardial infarction	Yes	116	X						X		X
#34	22	M	Not clear	Yes	120	X						X	X	X
#35	34	M	N.A.	Yes	120	X						X		X
#36	19	M	Acute cardiorespiratory decompensation	Yes	123	X	X	X	X	X	X	X	X	
#37	23	F	N.A.	Yes	125	X		X				X	X	
#38	22	M	N.A.	Yes	134	X						X		
#39	27	M	N.A.	Yes	136	X						X	X	X
#40	73	M	Cardiac decompensation with thrombotic occlusion of the descending aorta	Yes	138	X	X	X	X	X	X		X	
#41	68	F	Cardiac decompensation	Yes	141	X	X	X	X	X	X		X	
#42	26	F	Heart failure	Yes	168	X						X		
				No. of analyzed samples		40	15	17	17	16	16	25	26	14

Abbreviations: *B* = bone; *BL-A *= blood collected from autopsy; *BL-EMS* = blood collected by the EMS; *C* = cartilage; *EMS* = emergency medical service; *F* = female; *FC* = fibrous connective tissue; *H* = heart; *K* = kidney; *L* = lung; *M* = male; *N.A.* = not available; *SKM *= skeletal muscle

### Inclusion criteria for autopsy samples

We included individuals who died, the time of death was known, and an autopsy was performed. As an autopsy subgroup fatal OHCA cases were included to compare biomaterial collected by the EMS at scene with samples collected during autopsy.

### Blood samples for experimental testing

We also analyzed under experimental conditions blood samples isolated from 20 anonymous blood donors to reveal the effects of different storage conditions on the DNA stability. Blood was incubated up to 2 weeks at different temperatures and time intervals. Incubation was carried out at 4 °C, room temperate (RT), 32 °C, and 42 °C, and aliquots for gDNA isolation were frozen at − 22 °C at days 0, 1, 2, 3, 4, 5, 7, 10, and 14. For each time point, five biological replicates were analyzed. Five samples of the blood donors were also analyzed for DNA stability after repeated freeze-thaw cycles (0, 3×, 6×, 9×, 12×; see Supplements sFig. 1). All blood samples were collected in ethylenediaminetetraacetic acid (EDTA) containing plasma tubes (S-Monovette^®^, Sarstedt, Nümbrecht, Germany). Autopsy specimens were directly frozen on dry ice during examination. Samples included here were not used for genotyping in this study.

### Extraction of gDNA from biomaterials

The gDNA was extracted using the *High Pure PCR Template Preparation Kit* (Roche, Mannheim, Germany) according to the manufacturer’s instructions. To isolate gDNA from tissues, 30 mg (w/w) of specimens were incubated overnight with lysis buffer and Proteinase K at 55 °C. The gDNA was eluted in 50 μl elution buffer and stored at − 20 °C for further analysis. The concentration was measured with the NanoDrop 2000. The technical scatter of gDNA isolation on the DNA integrity was analyzed in triplicate.

### Determination of DNA integrity

DNA integrity was analyzed using the TAPEStation2200 (Agilent Technologies, Santa Clara, CA, USA). DNA stability was given as DNA integrity number (DIN) ranging from 0 to 10 with 10 representing no degraded gDNA. Approximately 5–150 ng/μl of gDNA was applied on a Genomic DNA ScreenTape (Agilent Technologies, Santa Clara, CA, USA) according to manufacturer’s instructions. The DIN is calculated by an automated algorithm, which determines the fragmentation of a gDNA sample by assessing the distribution of signal across the size range [17]. We regarded a DIN of 7 as a cutoff value for reliable NGS as recommended by the manufacturer [17].

To estimate the technical and biological scatter, we isolated gDNA in triplicates of three tissue specimens and determined the DINs by TapeStation analysis.

### PCR amplification of specific target sites in selected cardiac genes

We further validated the DIN analysis by PCR amplification of gDNA with different integrity values [18]. For PCR fragment analysis, single amplicons of four different genes were used (*NEXN*, *TTN*, *TNNI3*, and *MYL2*). These genes may carry DNA variants in genetic forms of cardiomyopathies and are located at chromosomal sites known to be sensitive for hydrolytic DNA cleavage [19]. PCR amplicons of these chromosomal sites were designed with different amplicon sizes (for MYL2-Ex.6 it was 206bp, TNNI3-Ex.7 381bp, TTN-Ex.27.1 430bp, and NEXN-Ex.12 529bp) for parallel analysis in one lane (primer sequences are available upon request).

### Statistics

For statistical analysis GraphPad Prism v9.4.1 for Mac was used (GraphPad Software, San Diego, CA, USA; www.graphpad.com). Medians are given within the manuscript including the upper and lower quartiles. In box and whiskers plots, boxes represent the quartiles and whiskers extend from 10 to 90 percentiles. Statistical significance was analyzed for multiple comparisons by Kruskal-Wallis or pairwise by Mann-Whitney test for non-Gaussian distributed data, where appropriate.

## Results

### Extraction of gDNA

**Fig. 3:**
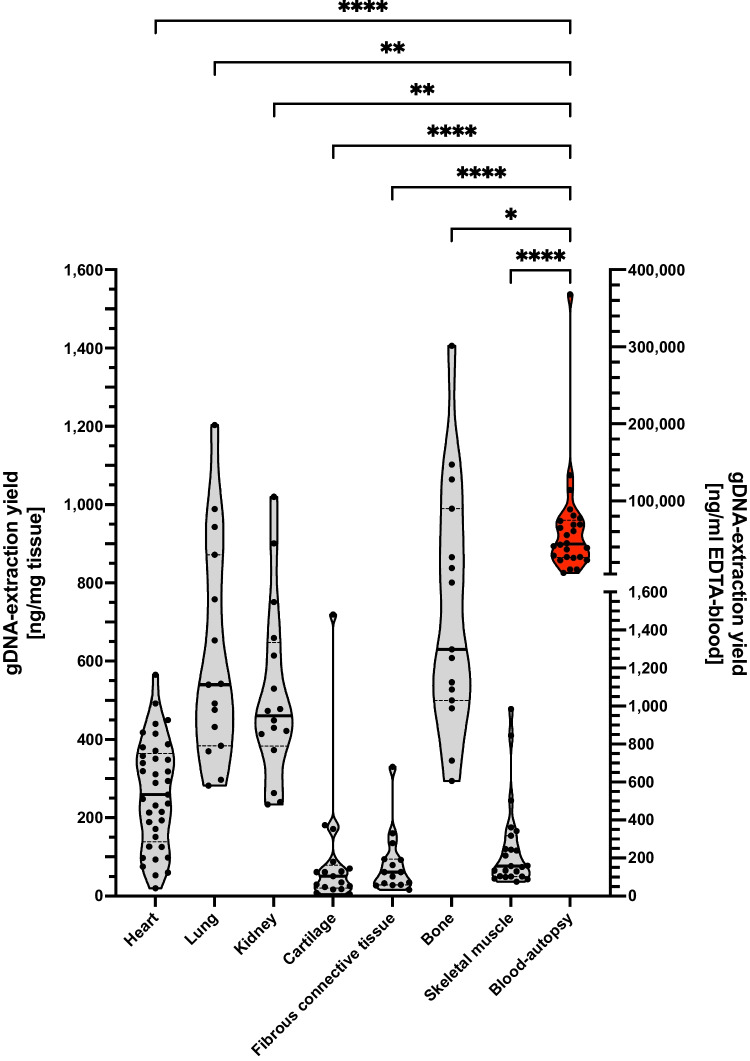
Violin plot of gDNA extraction yields of solid tissues (left *Y*-axis) and blood (red, right *Y*-axis) derived from autopsies. gDNA isolated from autopsy blood provides by far the highest yields of gDNA (Kruskal-Wallis test for multiple comparisons; medians are given as solid, quartiles as dotted lines)

We included biomaterial for DNA integrity measurements of 42 autopsy cases with a median age at death of 39 years (range 2–77 years; Fig. 2; Table 1). The median PMI at autopsy was 64 h (range 14–168 h; Fig. 2). The yield for gDNA extraction was highest in samples from blood (median 44; 26.8–74.9 μg/mL) and lowest in fibrous connective tissue (median 61; 29–94 ng/mg tissue) given that blood has a specific weight of 1.05 mg/mL [20] (Fig. 3; Table 2). Repeated freeze-thaw cycles had no influence on the DNA integrity (see sFig. 1).

**Table 2:** gDNA extraction yields and DIN derived from different autopsy tissues or EMS blood, respectively

Case no.	H		L		K		C		FC		B		SKM		BL-A		BL-EMS	
	gDNA yield*	DIN	gDNA yield*	DIN	gDNA yield*	DIN	gDNA yield*	DIN	gDNA yield*	DIN	gDNA yield*	DIN	gDNA yield*	DIN	gDNA yield**	DIN	gDNA yield**	DIN
#01	213	6.6											45	7			36,250	8.1
#02	60	8.1											65	7.6	69,250	7.4		
#03	171	5.8											49	7.4			47,750	8.8
#04			384	6.1	530	4.4	35	6.1	28	2.5	1,102	6.7			81,000	6.8		
#05	415	6.3																
#06	N.A.	7.5															19,000	8.1
#07	371	6.9	540	6.3	373	6.7	70	7.1	50	7.1	866	6.9			26,000	7.1		
#08	53	7.2											118	7			17,000	7.8
#09	492	3.8	758	6.1	240	1.9	8	6.9	330	5	1,064	7.2			43,000	6.5		
#10	231	2.1	476	6.6	263	2.3	23	6.4	79	6.8	1,406	6.9	116	6.9	69,000	7.4		
#11	388	5.9											166	6.6	55,500	1		
#12	358	6									N.A.	6.4	244	6.9	133,000	6		
#13	418	6.2	989	6.6	478	4.8	719	7.6	61	6	990	7.7			45,000	1		
#14	348	6	542	5.7	430	7.1	61	6.4	61	6.6	608	6.7			37,000	7		
#15	193	7.5											74	6.2				
#16	259	6.7	872	6.9	659	5.6	171	7.3	33	6.6	838	6.6			11,000	3.3		
#17	126	6.9											77	8.1			21,750	7.8
#18	75	7.8											43	7.9			40,500	7.8
#19	565	5.8	492	6.4	751	6.3	17	7.5	35	6.9	500	7.9			23,000	6.5		
#20	318	5					181	7.3	N.A.	6.4			410	7.2	29,000	6.2		
#21	215	6.9					63	8.1					175	8.2	114,000	7		
#22	248	6.6	653	5.7	414	2.5	18	6	94	7.8	528	6.1			11,000	6.2		
#23	319	6.3					5	N.D.					154	7.5	89,000	5.9		
#24	294	6.3											76	7.2			19,750	7.3
#25	151	6															40,000	8.4
#26	189	6.3											50	7.4	6,500	1	13,750	7.4
#27	93	1.4											478	6.7			18,250	7.1
#28					234	6.2									77,500	6.8		
#29	450	4.9	432	6.5	901	6.3	61	7	160	4	294	8.1			65,000	7.8		
#30	236	5	297	6.4	449	6.4	30	2.9	16	1.8	630	6.4			27,000	7.3		
#31	98	5.8											37	6.9				
#32	311	7.7	282	6.8	422	6.5			29	6.3	546	7.8			74,000	8		
#33	125	6.4											65	7.5			5,500	3.9
#34	351	6.4											120	7.2	60,750	6.1	5,500	7.1
#35	97	7											51	7.2			24,250	6.7
#36	340	2.8	943	6.6	473	2.5	51	6.3	27	6	480	6.6	103	6.9	27,000	6.6		
#37	N.A.	7.6			N.A.	6.9							N.A.	7.9	41,250	2.3		
#38	20	6.9											50	7.3				
#39	440	6.1											63	8	22,750	6.9	24,500	6.7
#40	380	6.3	1203	7.3	1020	6.7	87	6.5	135	5	801	6.8			368,000	2		
#41	289	6.5	370	6.1	614	6.3	26	5.8	92	6.4	346	3.9			39,000	6.9		
#42	N.A.	6.9											N.A.	8.5				

Abbreviations: *B* = bone; *BL-A* = blood collected during autopsy; *BL-EMS* = blood collected by the EMS; *C* = cartilage; *DIN* = DNA integrity number; *EMS* = emergency medical service; *FC* = fibrous connective tissue; *H* = heart; *K* = kidney; *L* = lung; *N.A.* = not available; *N.D.* = not detectable; *SKM* = skeletal muscle

*DNA yield in ng per mg tissue

**DNA yield in ng per mL blood

### The DNA integrity does not directly depend on the postmortem interval

The mean technical scatter for TapeStation analysis was 3.6% and 4.5% for combined gDNA isolation and TapeStation analysis (Supplements, sFig. 2).

**Fig. 4:**
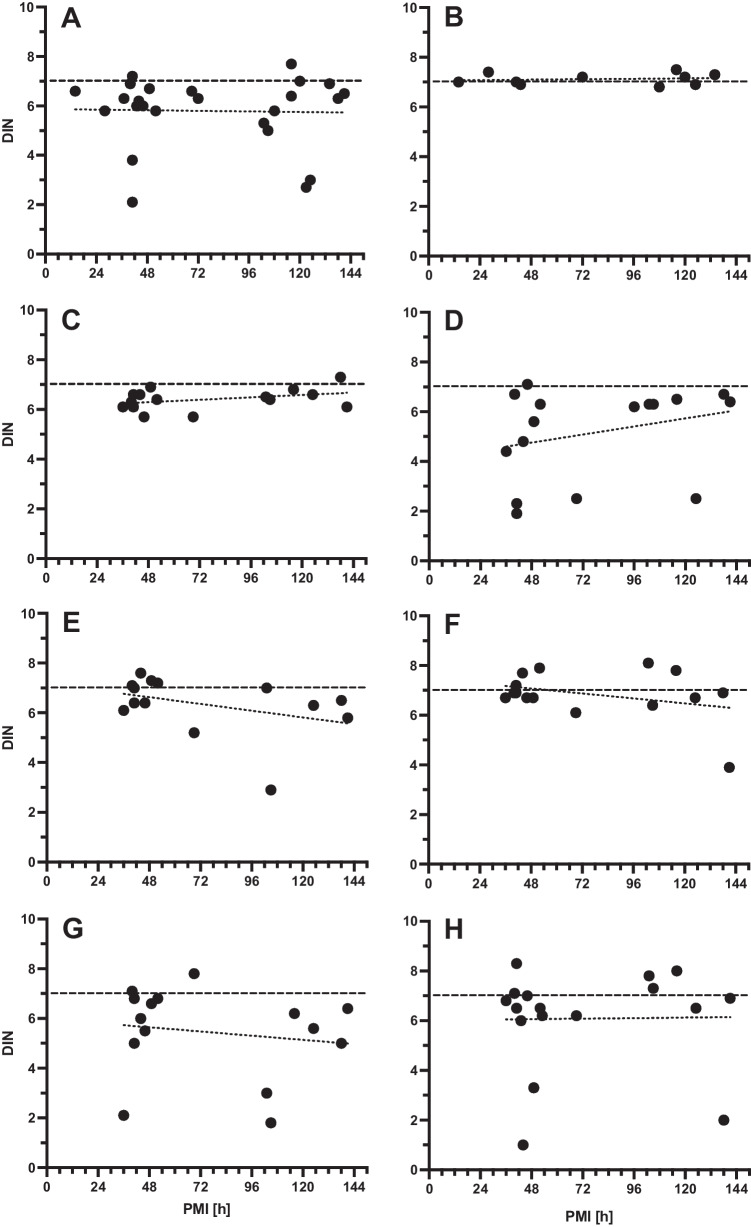
DNA integrity plotted against the postmortem interval (PMI) for different tissues from autopsy biomaterial. There is no linear correlation between DNA integrity and PMI (maximum 6 days). Results from linear regression analysis are given as dotted lines (slopes are not significant from zero). Dashed lines indicate cutoff of DIN = 7. **A** Heart; **B** skeletal muscle; **C** lung; **D** kidney; **E** cartilage; **F** bone; **G** fibrous connective tissue; **H** blood. DIN relative DNA integrity number

We correlated the DNA integrity against the PMI in different tissues from 42 autopsies. We measured the DIN in specimens from 40 hearts, 15 lungs, 17 kidneys, 17 cartilage tissues, 16 fibrous connective tissues, 16 bones, 25 skeletal muscles, and 26 blood samples. As a reference, we measured the DIN also in blood samples of 14 EMS cases (for details, see Table 2). We found that the DNA integrity is not directly dependent on the PMI but differs considerably between autopsy cases. However, samples derived from skeletal or cardiac muscle appeared to have the lowest scatter of DIN, as compared to all other tissues (Fig. 4). We did not find a correlation between PMI and DIN in autopsy samples by linear regression analysis (Fig. 4). We also compared the DIN of different tissues within the same autopsy case with considerable differences between tissues and PMI. Thus, the DIN depends in an unpredictable manner on the case, the tissue, and the PMI (Supplements, sFig. 3).

### gDNA isolated from blood samples collected by the EMS has the highest integrity

**Fig. 5:**
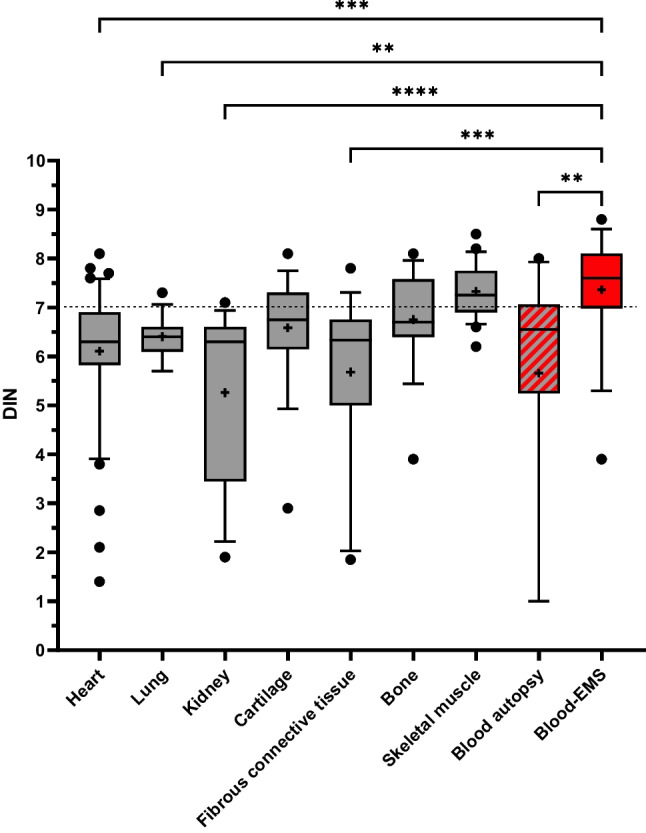
Comparison of the DNA integrity number (DIN) derived from autopsy samples including blood and blood samples collected by the emergency medical services (EMS). The DIN of the EMS blood samples from 14 fatal OHCA cases is significantly higher (*p* = 0.005) as compared to any other tissue except cartilage, bone, and skeletal muscle from autopsy. For the statistical evaluation, the Kruskal-Wallis test for multiple comparisons was used (box and whiskers plots: boxes represent the quartiles, whiskers extend from 10 to 90 percentiles, outliers are given as dots, medians as solid lines and means as crosses)

We compared the DNA integrity of all biomaterials collected during autopsy and by the EMS (Table 2; Fig. 5). The DIN of blood samples from autopsy (BL-A; median 6.6; 5.3–7.1) and from the EMS (BL-EMS; median 7.6; 7–8.1) were significantly different (Kruskal-Wallis test, *p* = 0.005). The 25% percentile of the BL-EMS DIN was 7.0 and above the level recommended for NGS analysis. Vice versa 75% of BL-A samples were at or below the cutoff recommended for NGS sequencing (Fig. 5) [17].

Notably, among the autopsy biomaterials tested in this study skeletal muscle revealed the highest levels of DIN, which was significantly higher as compared to samples from cardiac, renal, and fibrous connective tissues (Fig. 5).

### EDTA blood samples from blood donors provide intact gDNA even for several days under robust storage conditions

We further compared blood samples collected during autopsy and from blood donors under experimental conditions. DINs of samples collected from blood donors plotted against the storage time could be fit by linear regression analysis independently of the storage conditions, revealing degradation of DNA integrity over time (slope significantly different from zero, *p* < 0.001; see Supplements sFig. 4). In contrast, DINs of blood samples from autopsy plotted against the PMI could not be fitted by regression analysis (slope different from zero in linear regression analysis, *p* = 0.967; see Supplements sFig. 4). This reveals that the stability of gDNA derived from autopsy blood samples cannot be predicted from the postmortem time.

**Fig. 6:**
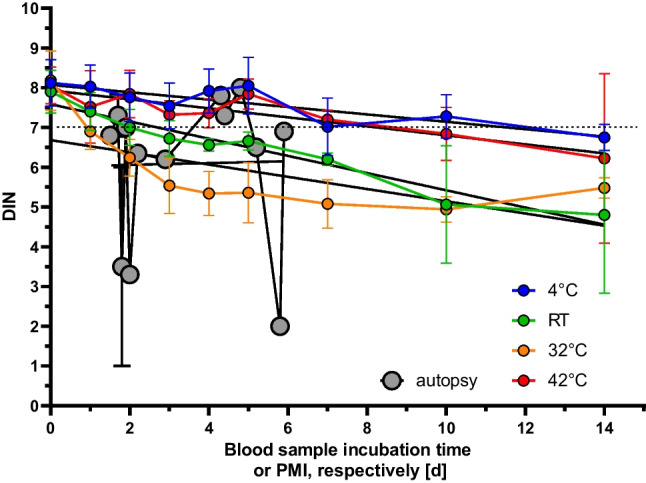
Experimental comparison of the influence of different storage conditions (4 °C, room temperature (RT), 32 °C and 42 °C) on EDTA blood samples from donors or of blood collected during autopsy on DNA integrity, respectively. The DNA integrity number (DIN) is plotted against the blood sample incubation time or postmortem interval (PMI). Blood samples from autopsy are shown as means ± standard error. For donor blood samples stored at different temperatures, the means ± standard deviation is given (see also Supplements sFig. 4)

We observed that the DIN of samples stored at room temperature (RT) remained above the cutoff of 7 for 2 days (Fig. 6). Even after storage of blood samples for 5 days at RT, the DIN was above the median of blood samples collected during autopsy (compare Fig. 5 and Fig. 6). The quality of gDNA could be improved storing the blood samples at 4 °C, yielding a median DIN of 7.6 (7.1–8.2; Fig. 6). The incubation of blood samples at 32 °C accelerated the degradation process, whereas the DNA integrity measured in samples stored at 42 °C was comparable to samples kept at 4 °C (Fig. 6), but the yield dropped considerably (Supplements sFig. 6).

### PCR works also with gDNA of low integrity

We found for all targets PCR amplicons, indicating that *targeted Sanger* sequencing may work even with fragmented gDNA (DIN range 1–7.7; see Supplements sFig. 5).

## Discussion

Sudden cardiac death in the young is a devastating event for relatives. For the EMS, it remains challenging to identify non-ischemic cardiac failure in patient with need for OHRA. It is known that the etiology for cardiac failure differs considerably with patient age. In a Danish nationwide study, coronary artery disease was the main cause of SCD in patients between 1 and 49 years [4]. In young cases of OHCA, a major cause of cardiac arrest may be associated with a considerable burden of genetic etiologies [21]. It is known that in fatal OHRA, the cause of death may be unraveled by autopsy. Autopsy without molecular analysis may fail to identify the cause of death in a considerable high number of cases, i.e., in patients with sudden arrhythmic death [22]. In addition, low DNA integrity due to postmortem decomposition processes can impact the option to perform routine high-throughput NGS. However, the parallel DNA sequencing of a high number of genes or even the whole exome in cases of young fatal OHCA is mandatory [23]. We found that DNA isolated from frozen tissue and even with low DIN can provide PCR amplicons suitable for Sanger sequencing. In families with known genetic predispositions, PCR-based Sanger sequencing may be performed in autopsy material even with low-quality DNA [24]. Whereas in fatal OHCA cases of unknown etiology, hundreds of genes must be sequenced and therefore Sanger sequencing will not be appropriate for molecular autopsy.

The ERC-2021 guidelines recommend consistently to consider an advanced post-resuscitation care including autopsy and molecular analysis of cardiovascular diseases in not precisely selected fatal OHCA cases [1]. In an ideal setting and according to the ERC guidelines, this combined procedure will have an impact on unraveling even rare forms of genetic diseases, which not necessarily have to be inherited in cases of de novo variants [25] or homozygous genotypes [26].

Unfortunately, the rate of autopsies in many countries is notoriously low for several reasons and the PMI may lead to gDNA degradation limiting the extent of possible molecular analysis. In a recent EMS study, about 40% of fatal OHCA cases were not transported to a hospital and about 43% of cases were not autopsied [16]. This shows that the ERC-2021 guidelines do not consider fatal cases who are not transported to a hospital and/or are not transferred to autopsy.

We recently suggested that the EMS has the unique opportunity in young fatal cases of OHCA to collect a blood sample at scene [8]. We showed that the number of fatal OHCA cases with an identified predisposition for cardiac arrest was remarkably higher when the EMS collects a blood sample on scene as compared to those cases identified by autopsy only [16]. For these reasons, the EMS should always save a blood sample for later molecular autopsy in young (1–50 years of age) fatal OHCA cases [16], which is currently not recommended by the ERC guidelines [1].

In EMS practice, it is important to know how stable a blood sample will be for a possible subsequent NGS analysis. Therefore, we analyzed in parallel samples from different tissues derived from autopsies and compared the data to the biomaterial, which was collected by the EMS. We found that gDNA from autopsy had significantly lower DNA integrities depending on tissue and PMI as compared to EDTA blood samples collected by the EMS. We also found that the yields and the DNA integrity differed among autopsy cases in an unpredictable manner, which is in line with problems to calculate the PMI from gDNA fragmentation in forensic medicine (for a review, see [27]). The best DNA integrity but with a low extraction yield was found in samples of the skeletal muscle, which is in concordance with previous work [9, 12, 28], whereas the blood samples collected during autopsy delivered quite heterogenous results.

We also measured the DNA integrity of EDTA blood under challenging experimental storage conditions, simulating real-life conditions of blood sample collection by the EMS. The integrity of the DNA is not influenced by repeated freezing-thawing revealing stability of the genomic DNA under robust handling conditions (see sFig. 1). The gDNA degradation is enzyme dependent, which is reflected by the temperature dependency of its storage conditions. At experimental temperatures of 4 °C or 42 °C, the enzymatic degradation of gDNA is low in comparison to RT or 32 °C, respectively. Since DNA degradation is an enzyme catalyzed process, gDNA remains stable at higher temperature, but the extraction yield at 42 °C was considerably low (Supplements sFig. 6). However, EDTA blood samples incubated for even up to 10 days at 4 °C delivered gDNA with a DIN at 7. Even storage at RT will provide DNA stability for up to 2 days revealing the feasibility of sample handling by the EMS personnel. This further indicates that blood sample collection by the EMS will provide excellent biomaterial for later molecular autopsy—especially in fatal cases of OHCA.

An interdisciplinary network of pathologists, molecular biologists, cardiologists, and geneticists is most effective for unraveling genetic causes of fatal cardiac arrest. However, this network will be most effective by integrating the EMS. The EMS is essential to unravel the medical circumstances of death in fatal OHCA, who are not transported to a hospital and/or not autopsied. Thus, with little efforts, the identification of genetic forms of severe cardiovascular diseases with fatal outcome can be improved.

## Conclusions

1. Our data show that in this study, the integrity of gDNA in blood samples collected by the EMS is higher as in any biomaterial from autopsy. We recommend changing future ERC guidelines accordingly: in fatal cases of young OHCAs, the collection of a sample of 2 mL EDTA blood is a safe and reliable way to obtain gDNA for later molecular autopsy, especially of those who are not transported to hospital. This requires no special storage or handling conditions. The collection of a 2 mL blood sample by the EMS should be mandatory in fatal OHCA cases younger than 50 years of age. An adequate blood sample ensures the option of a later targeted consultation of survivors and the relatives, when compared with clinical or pathological findings.
2. If no blood sample collected by the EMS is available and an autopsy is performed, specimen of the skeletal muscle should be considered for gDNA extraction. Since the yield of gDNA extraction is limited, in skeletal muscle, we recommend to isolate 1 cm^3^ tissue for immediate freezing (− 20 °C or lower). In any case, tissue must not be fixed by formalin if NGS is considered for subsequent investigation.

### Limitations

For the determination of DNA stability, we determined the DNA integrity number (DIN) using an established semiautomated chromatography method [17]. We did not perform NGS genotyping in parallel. DINs above 7 are recommended by the NGS manufacturer to generate reproducible sequencing data and therefore used as a technical cutoff in this study. Since the correlation of DIN and NGS data quality has a high scatter, DINs below 7 may nevertheless deliver acceptable sequencing results in selected cases.

## Supplementary information

ESM 1: Supplements. SFigs. 1–6 (DOCX 345 kb)

## Data Availability

The datasets analyzed during the current study are available from the corresponding author on reasonable request.

## Abbreviations

BL-A: Blood sample collected during autopsy
BL-EMS: Blood sample-collected by the emergency medical service
DIN: DNA integrity number
DNA: Desoxyribonucleic acid
EMS: Emergency medical service
EDTA: Ethylendiaminetetraacetic acid
ERC: European Resuscitation Council
FFPE: Formalin-fixed paraffin-embedded tissue
gDNA: Genomic DNA
NGS: Next-generation sequencing
OHCA: Out-of-hospital cardiac arrest
OHRA: Out-of-hospital resuscitation attempt
PCR: Polymerase chain reaction
PMI: Postmortem interval
RT: Room temperature
SCD: Sudden cardiac death
