# Supplementary material for: Blood taken immediately after fatal resuscitation attempts yields higher quality DNA for genetic studies as compared to autopsy samples
Source: Int J Legal Med. 2023 Feb 11;137(5):1569–81. doi: 10.1007/s00414-023-02966-7 (PMC10421769; doi:10.1007/s00414-023-02966-7)
Supplement: Supplementary file 1 — Supplements. SFigs. 1–6 (DOCX 345 kb) [file 414_2023_2966_MOESM1_ESM.docx]

**Supplements**

**sFig. 1.** The DNA-integrity number (**DIN**) of gDNA is not influenced by repeated freeze-thaw cycles.

**sFig. 2.** Technical reproducibility of gDNA isolation and DNA-integrity number (**DIN**) determination in 3 autopsy cases (#14, #27 and #36). Triplicate analysis of DNA-integrity. The standard deviation is 3.6% of the means, the relative standard deviation of gDNA isolation *and* DNA-integrity (marked ‘+’) accounts for 4.5%. Values are shown as means ± 95% CI.

**sFig. 3.** Comparison of the DNA-integrity number (**DIN**) in different tissues derived from autopsy. No. autopsy cases are shown on the right side, who were autopsied at different postmortem intervals: **A**) 2, **B**) 3, **C**) 5, **D**) 6 days PMI.

**sFig. 4.** Regression analysis of DNA-integrity number (**DIN**) against incubation time under different conditions or postmortem interval (**PMI**) of blood collected during autopsy. The slope of the linear regression analysis for blood samples incubated at experimental conditions is significantly different from zero (room temperature (**RT**), 4°C, 32°C p<0.0001, 42°C p= 0.0003). Fitting of autopsy data was not possible.


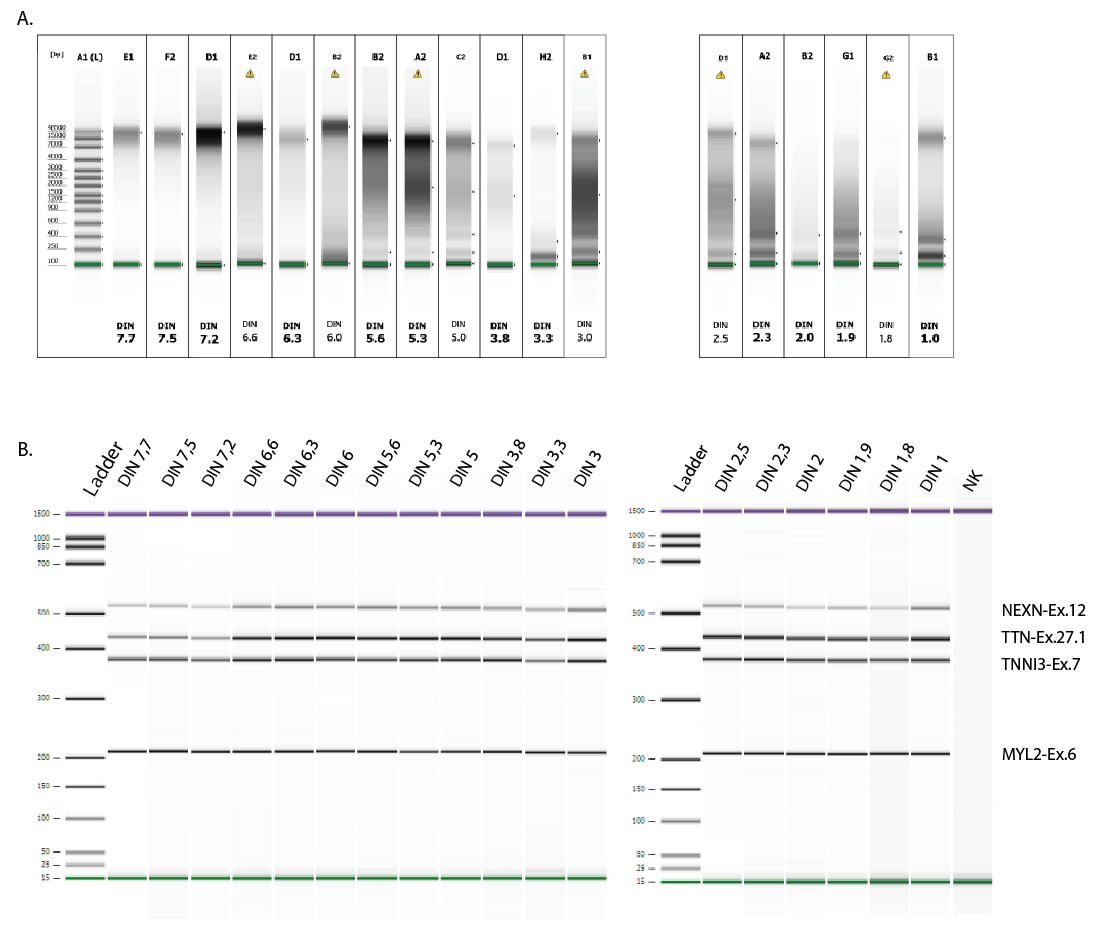


**sFig. 5.** PCR products of different target genes: ***NEXN*** exon 12, ***TTN*** exon 27.1, ***TNNI3*** exon 7 and ***MYL2*** exon 6 amplified in gDNA with different DNA-integrity numbers (**DIN**). **A**) Electrophoretic pattern of gDNA as a PCR template and corresponding DINs measured with TapeStation 2200. All samples were run under the same experimental conditions. The graphic of the gel has been edited for presentation. **B**) PCR fragments of different sizes derived from the four different genes shown at the right margin are separated in a gel matrix. Of note, PCR-fragments of the expected size are found in all DNA-preparations irrespective of the DIN.

**sFig. 6.** Linear regression analysis of gDNA-extraction yields from blood samples stored at different temperatures over time. gDNA isolation was done in 5 replicates. The yields decrease over time significantly for the blood samples incubated at 42°C (red line; linear regression, slope different from zero p<0.0001). **Abbreviations**: **RT**= room temperature, **SD**= standard deviation.
